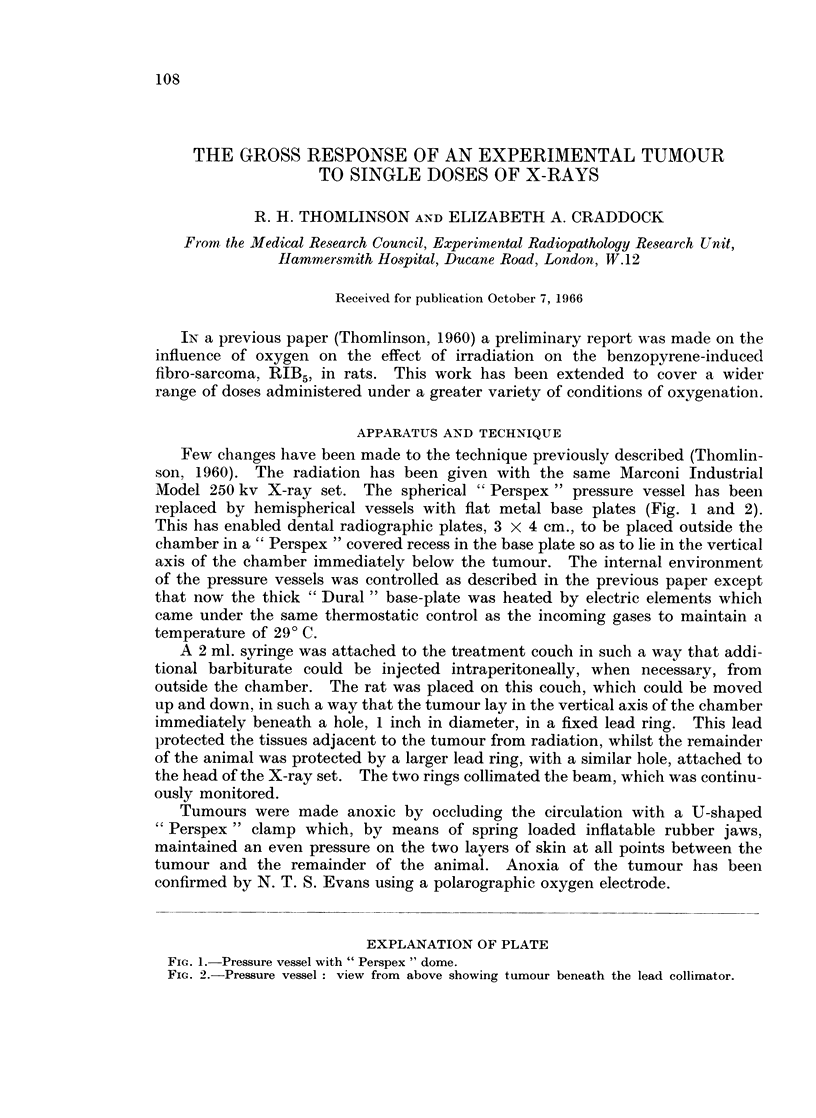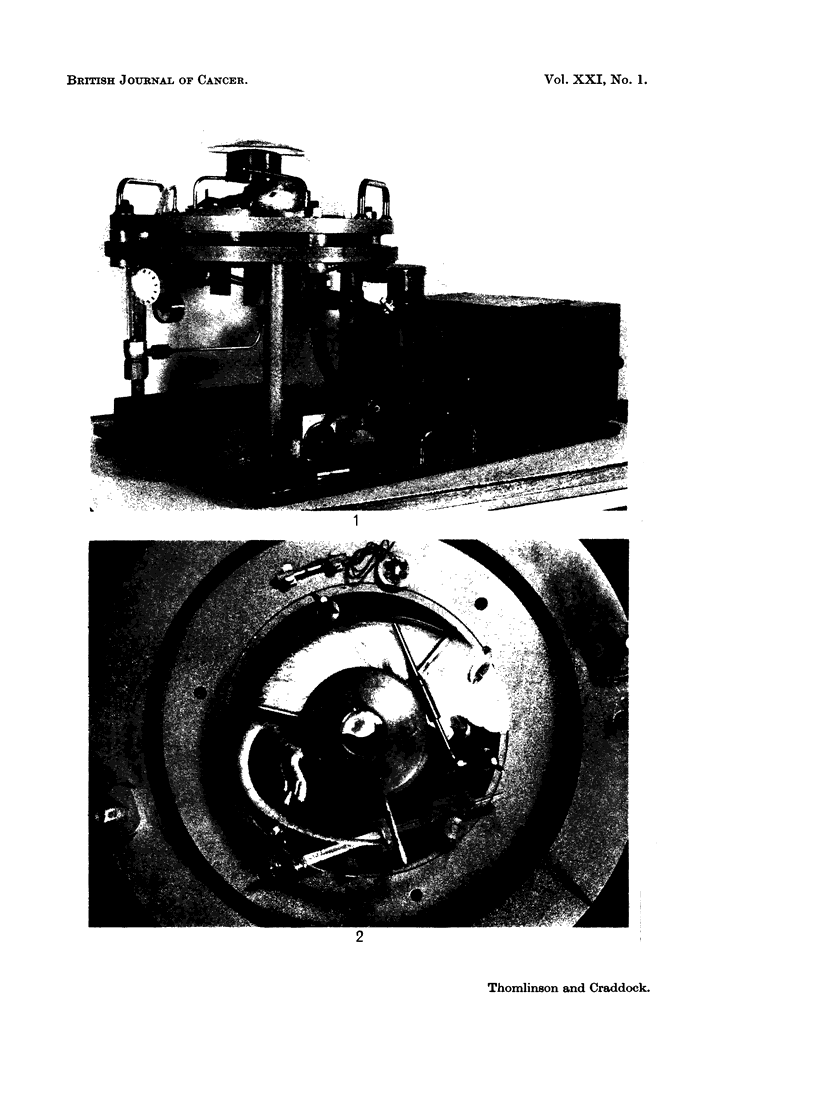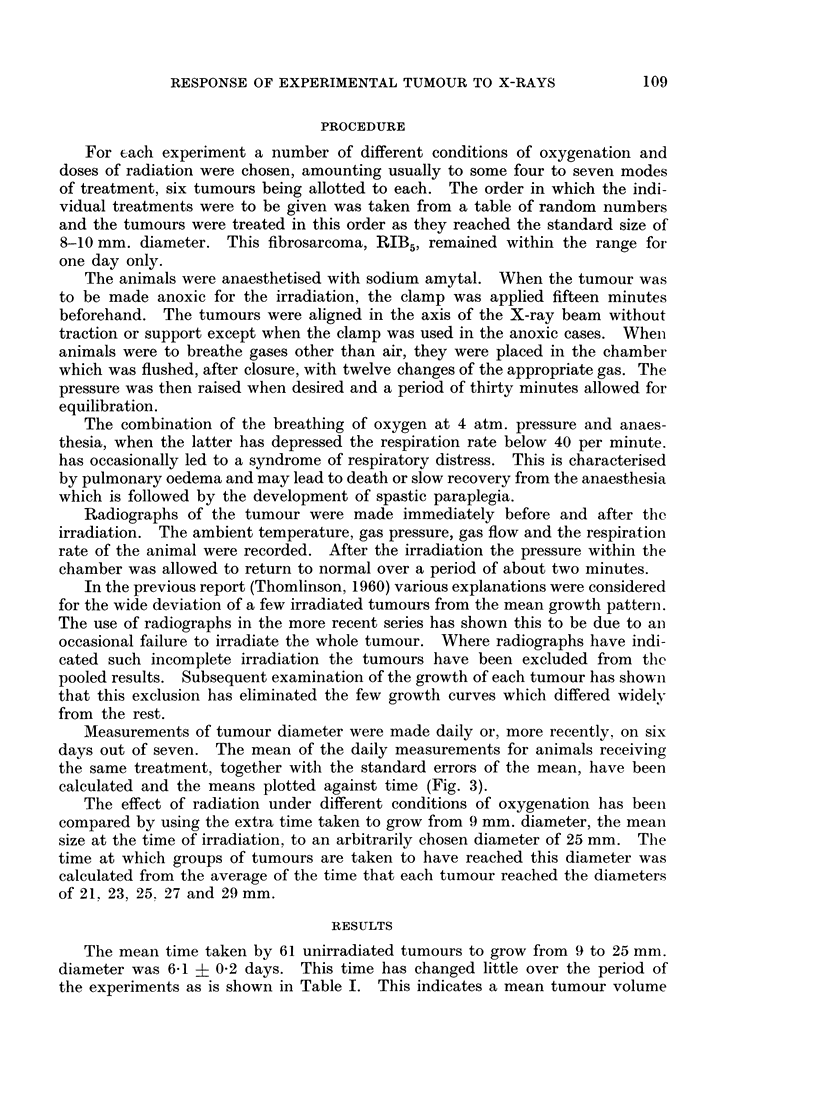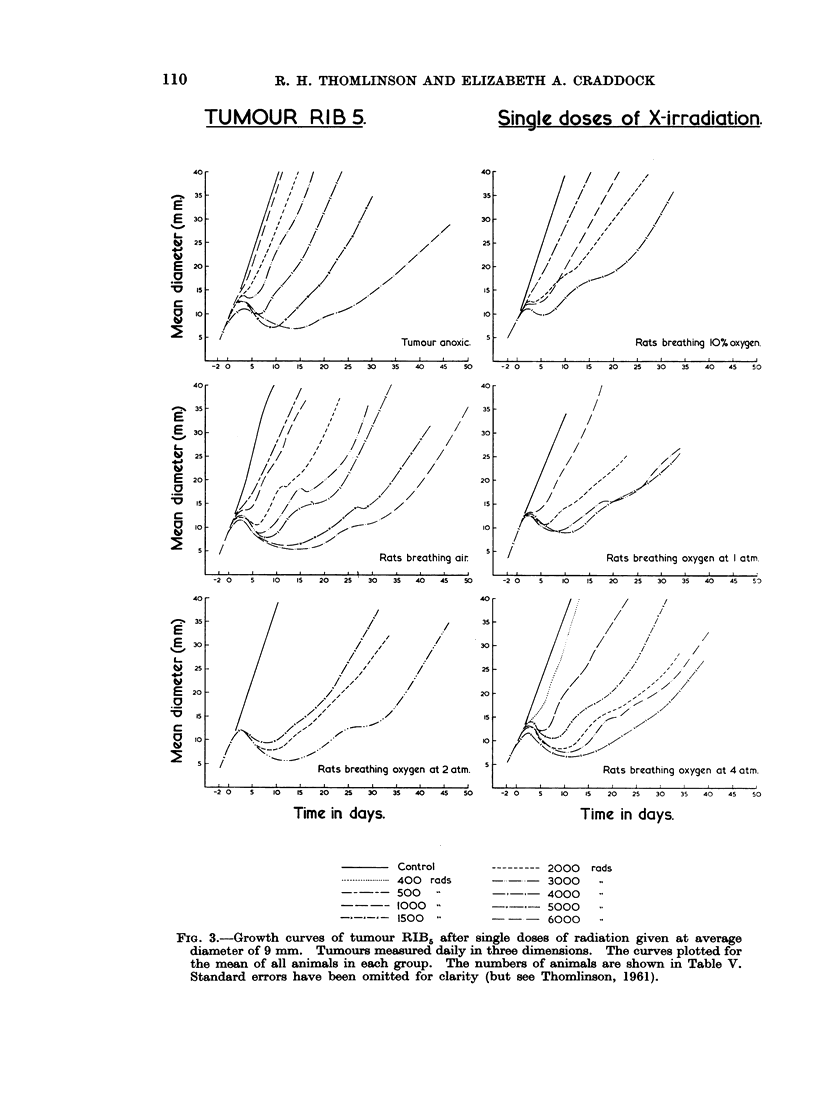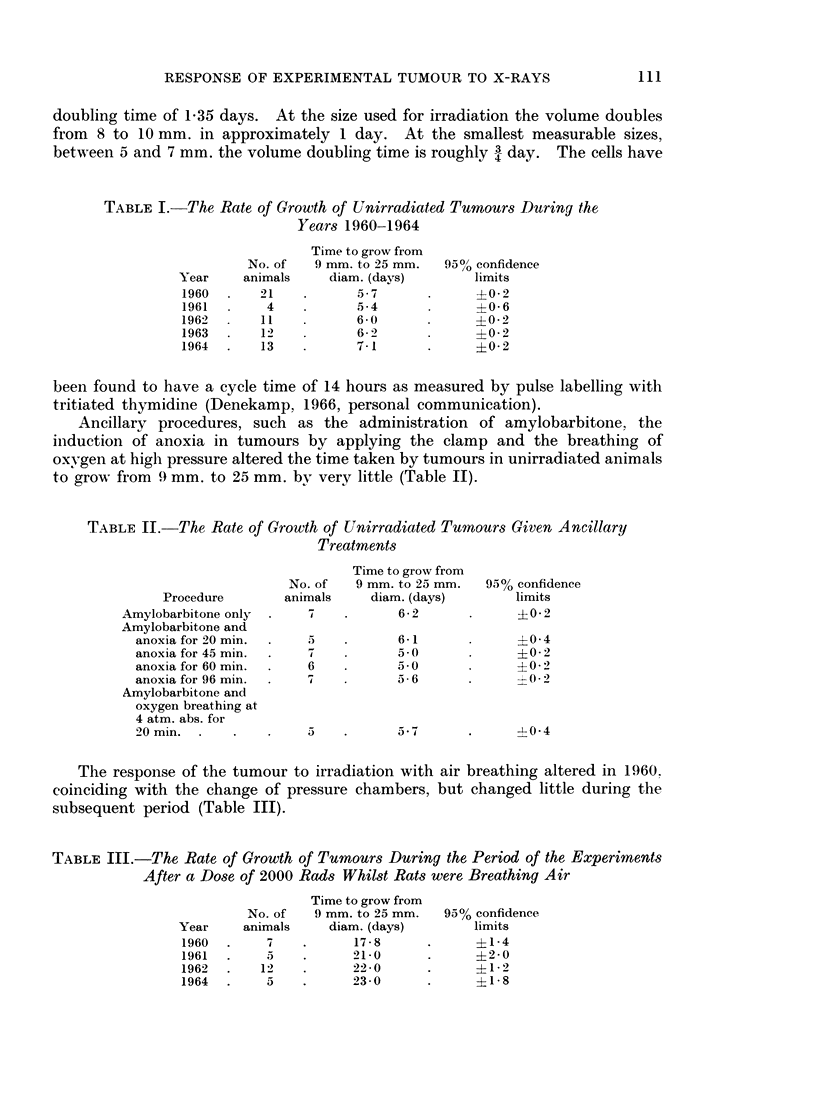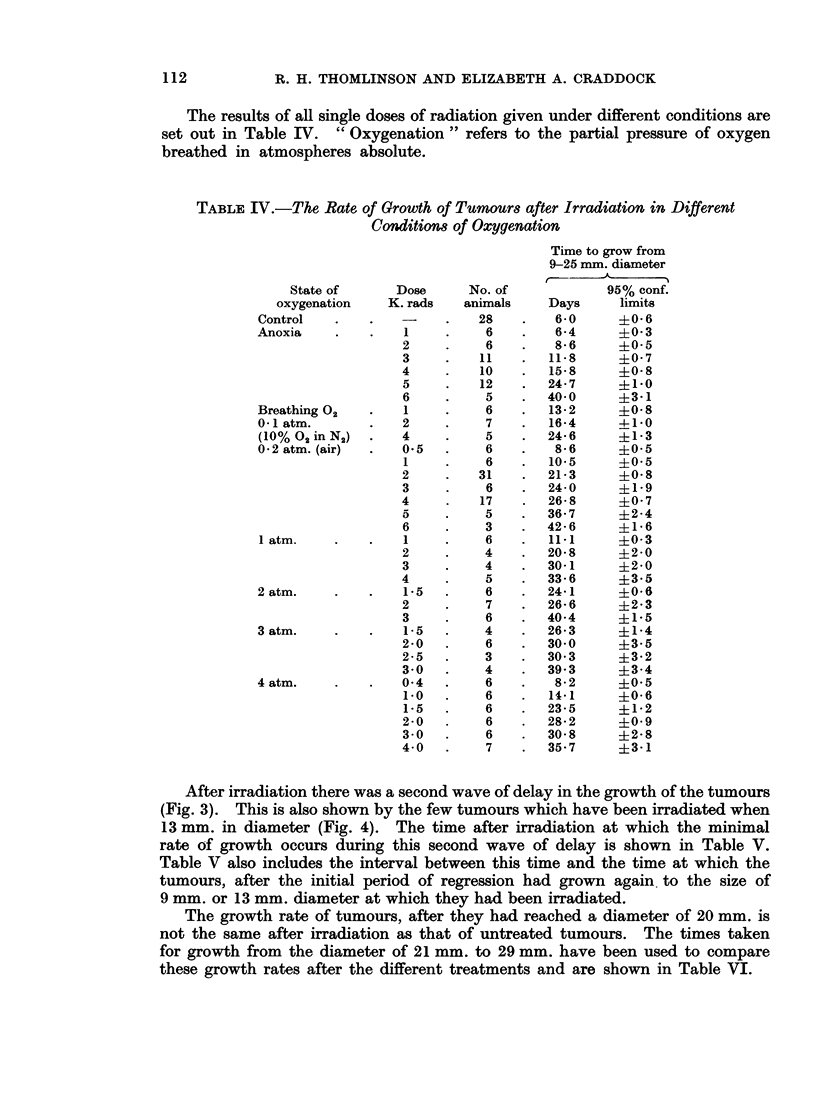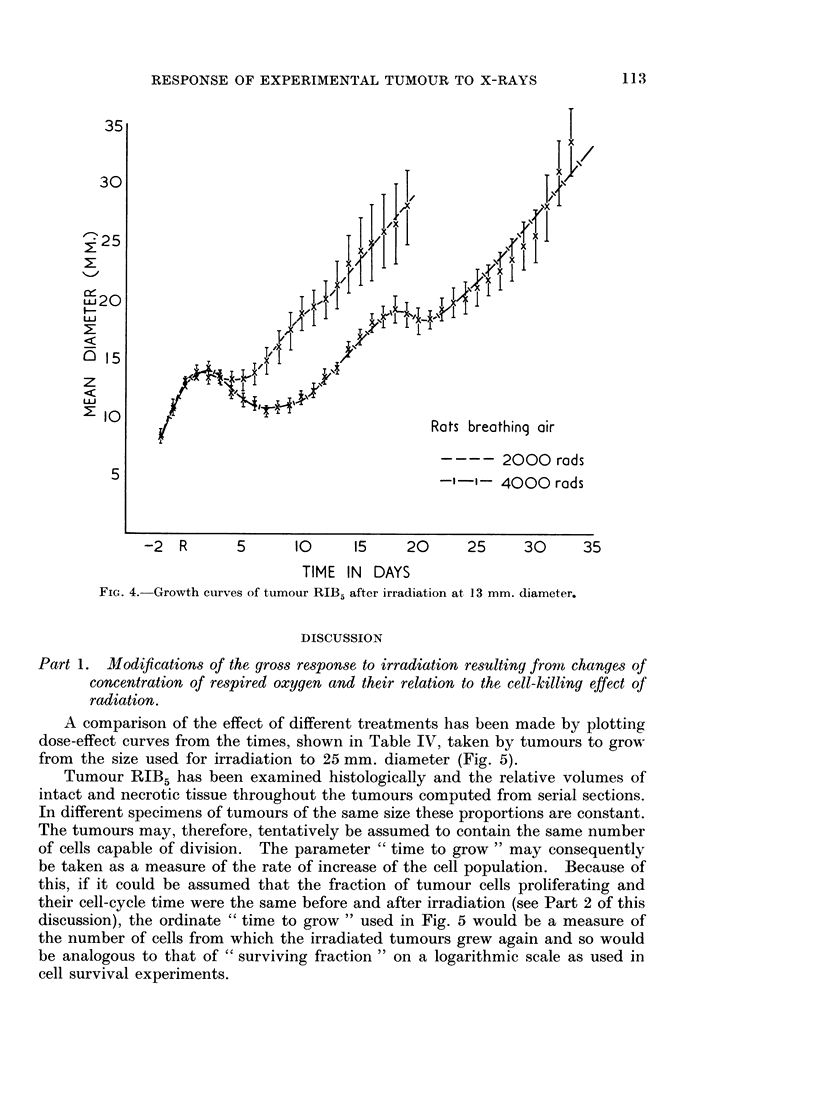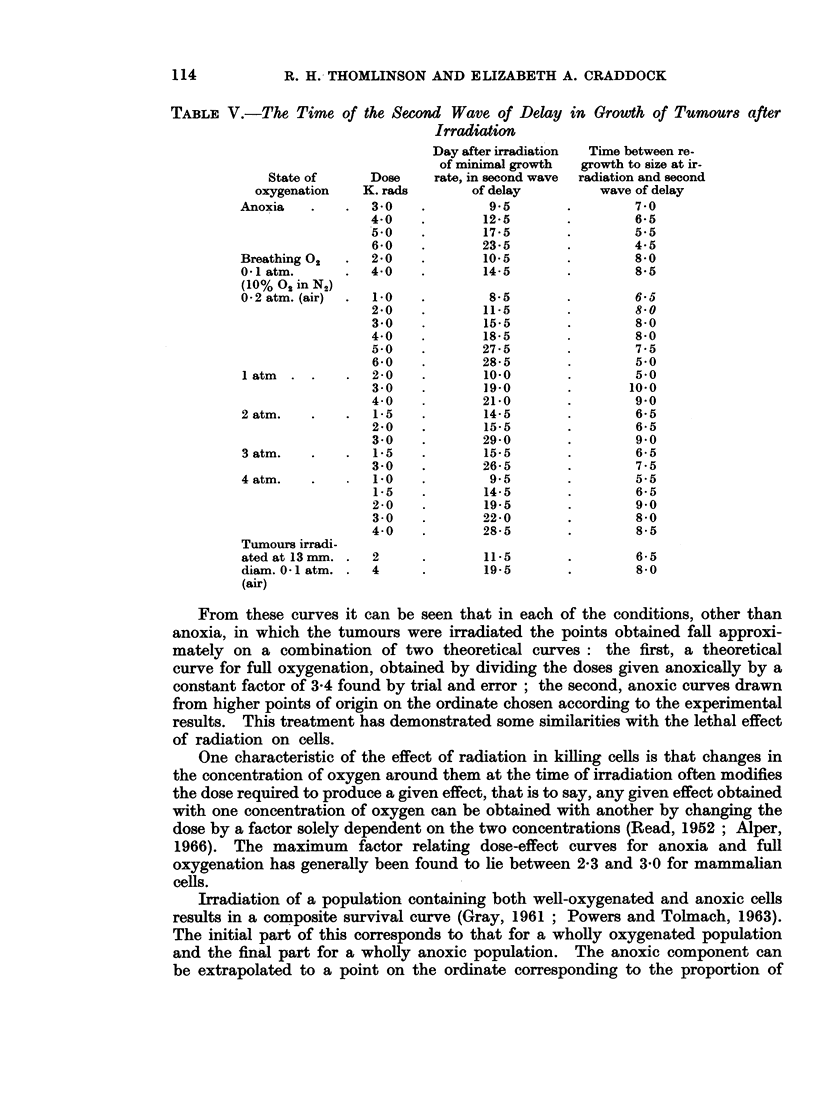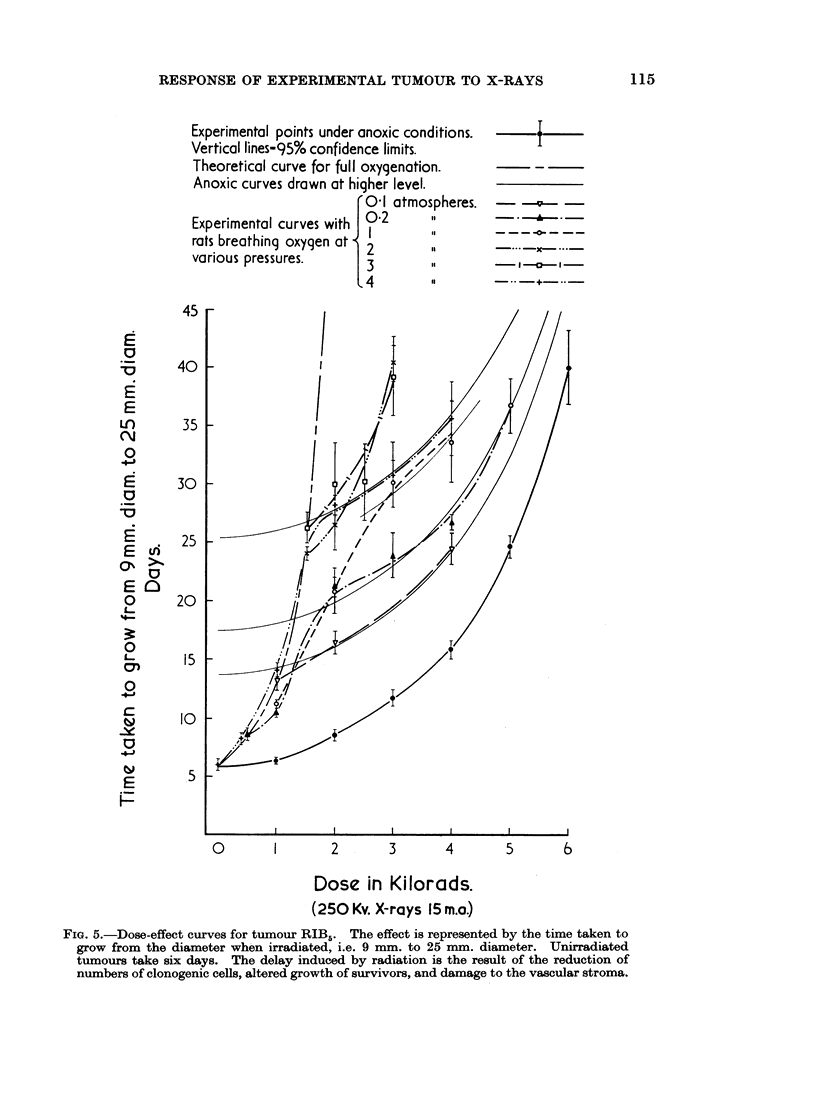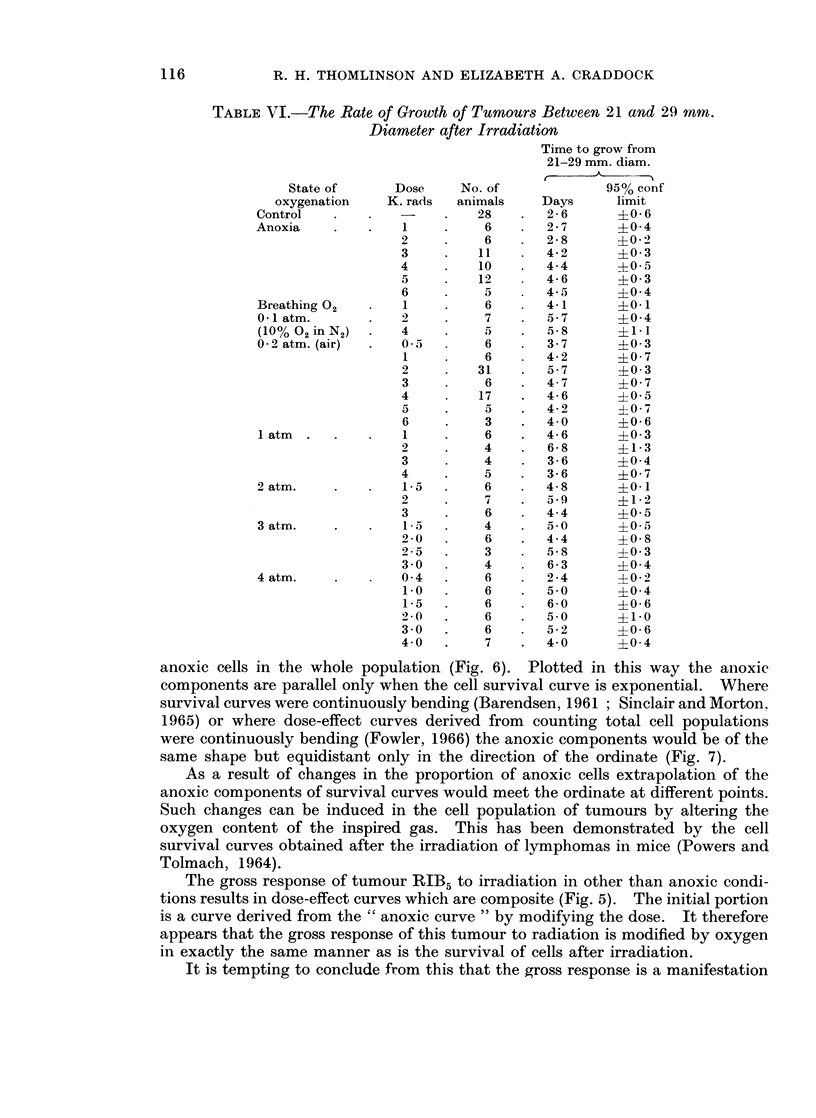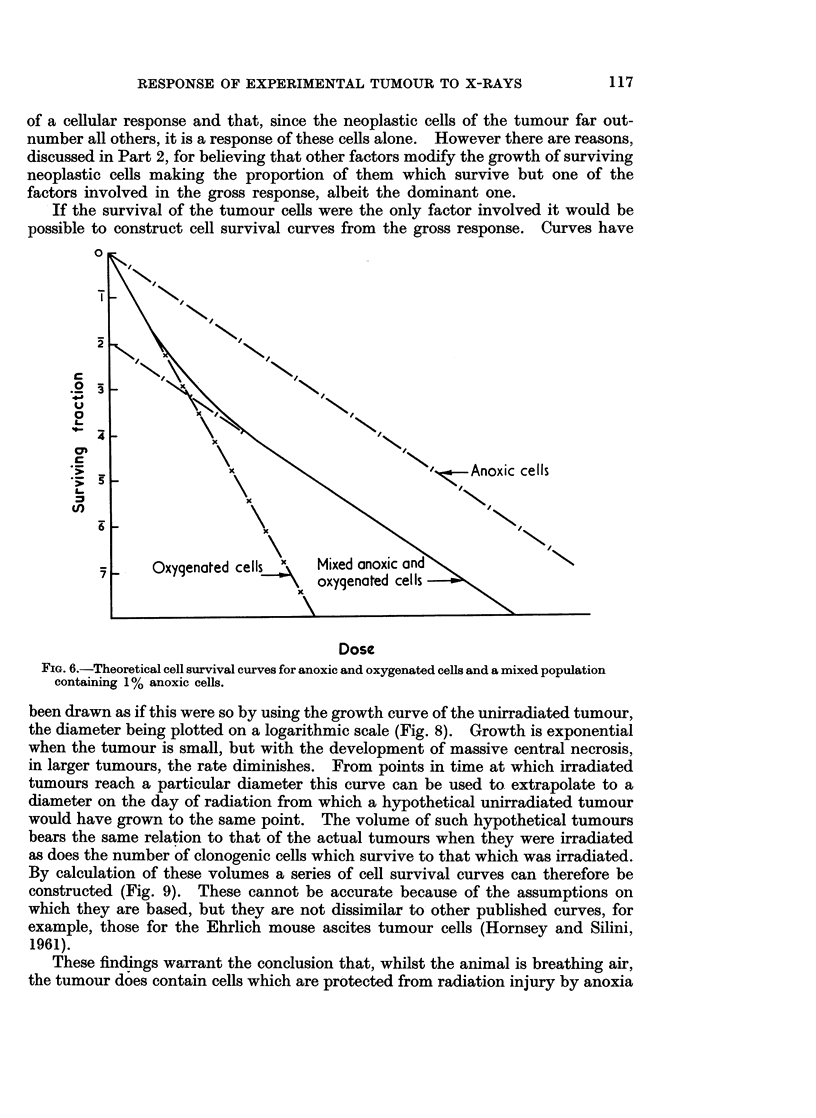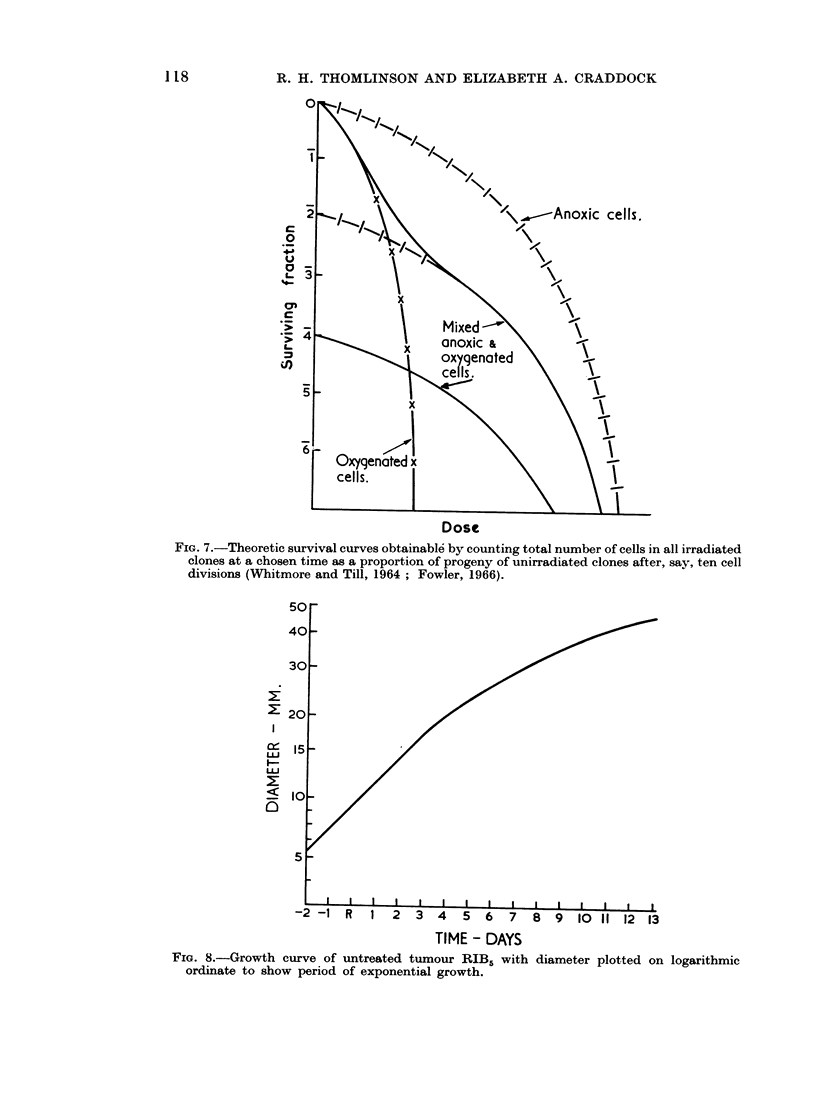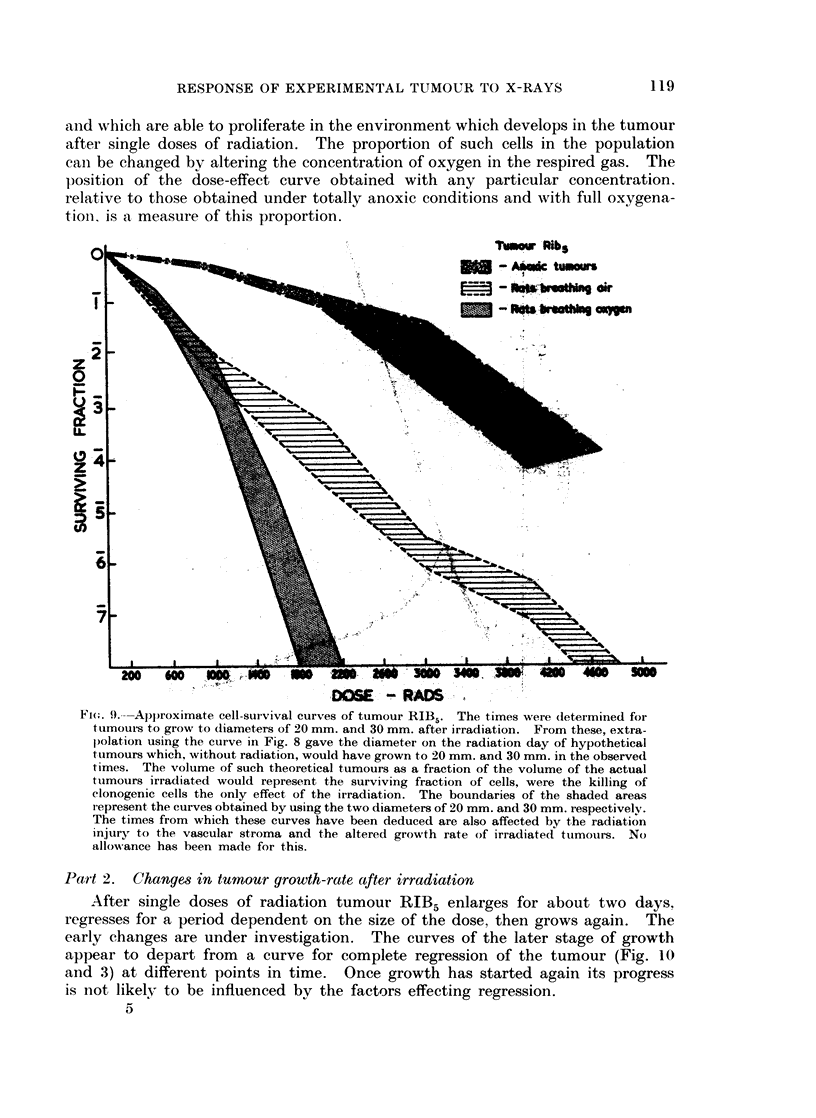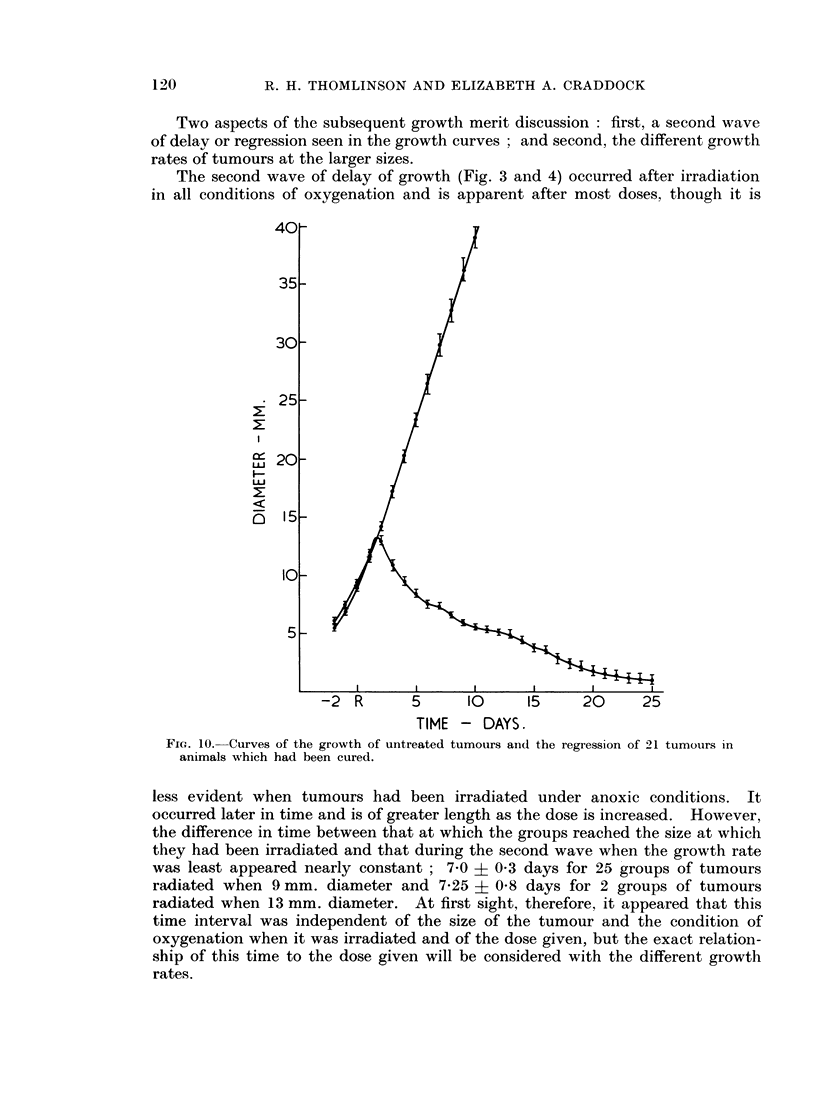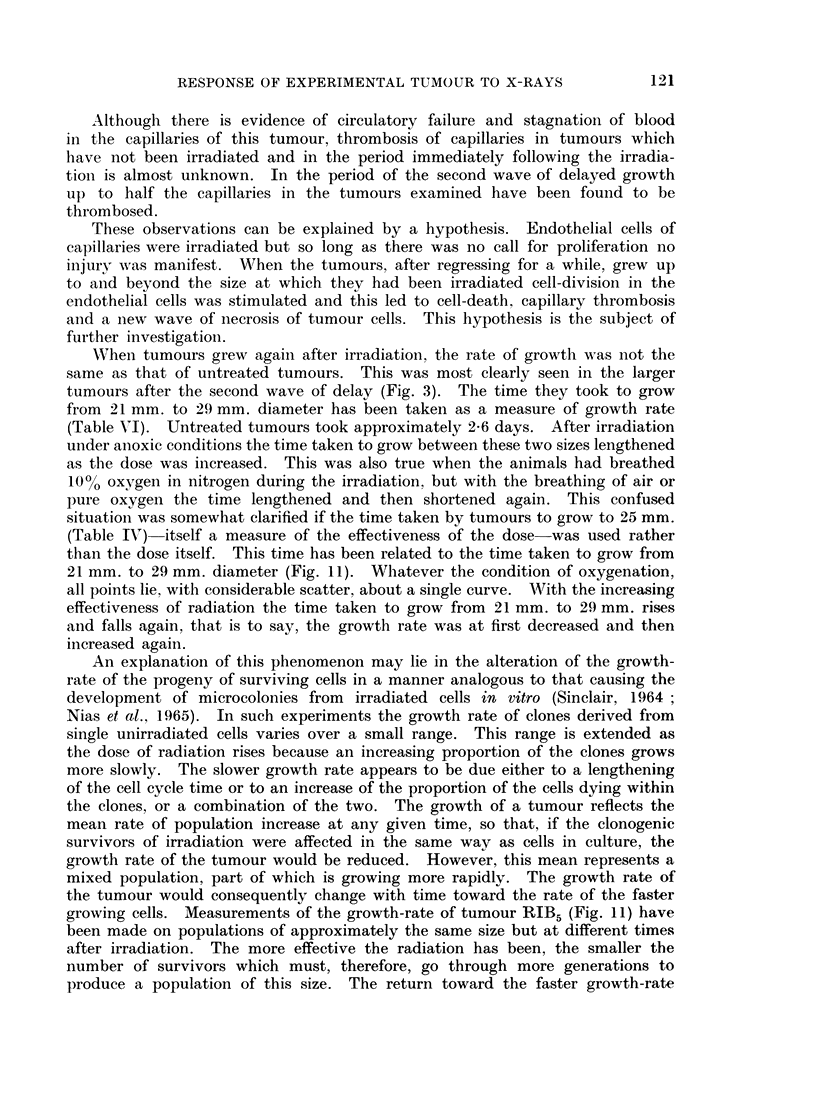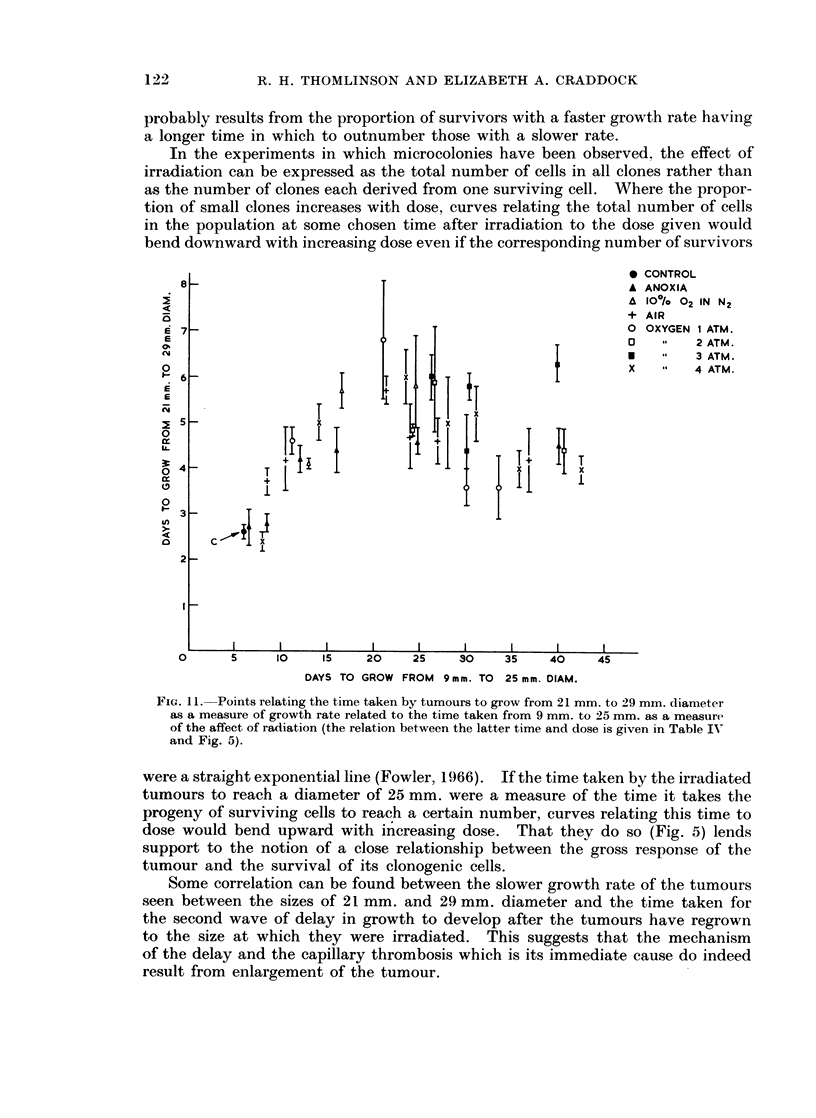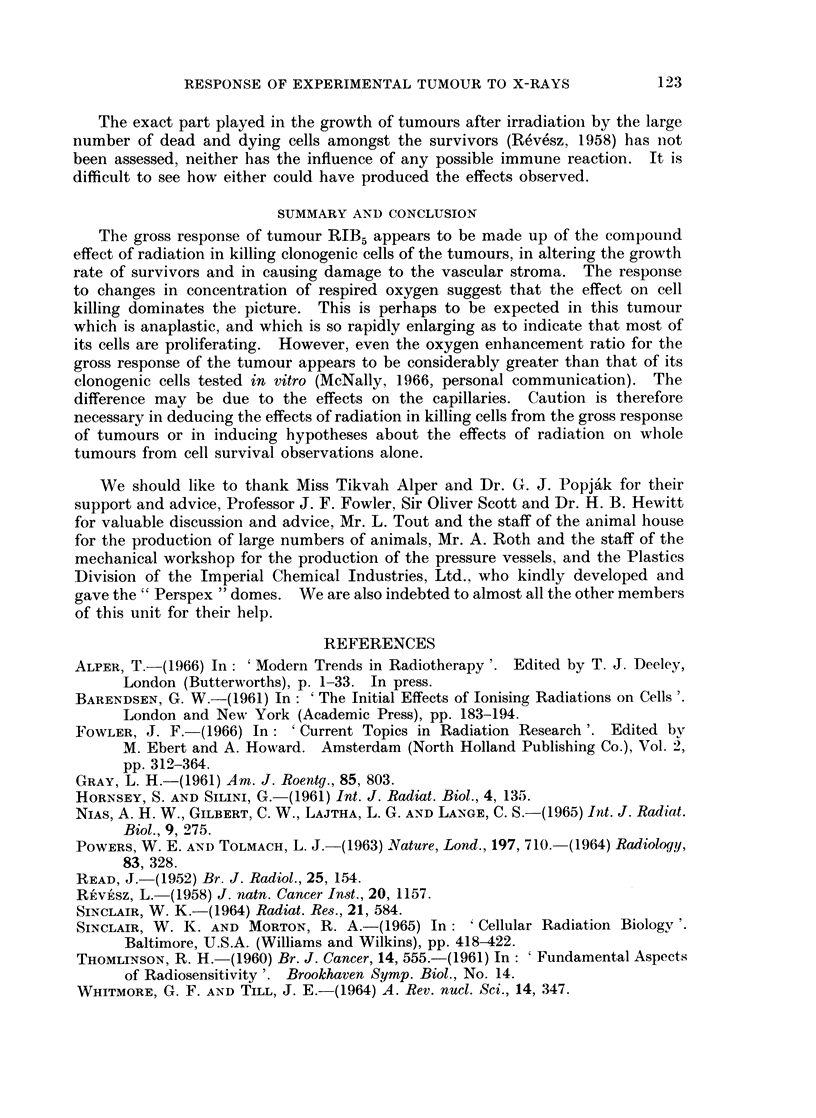# The gross response of an experimental tumour to single doses of x-rays.

**DOI:** 10.1038/bjc.1967.10

**Published:** 1967-03

**Authors:** R. H. Thomlinson, E. A. Craddock

## Abstract

**Images:**


### THE GROSS RESPONSE OF AN EXPERIMENTAL TUMOUR TO SINGLE DOSES OF X-RAYS

R. H. THOMLINSON AND ELIZABETH A. CRADDOCK

*From the Medical Research Council, Experimental Radiopathology Research Unit, Hammersmith Hospital, Ducane Road, London, W.12*

Received for publication October 7, 1966

IN a previous paper (Thomlinson, 1960) a preliminary report was made on the influence of oxygen on the effect of irradiation on the benzopyrene-induced fibro-sarcoma, $RIB_5$, in rats. This work has been extended to cover a wider range of doses administered under a greater variety of conditions of oxygenation.

### APPARATUS AND TECHNIQUE

Few changes have been made to the technique previously described (Thomlinson, 1960). The radiation has been given with the same Marconi Industrial Model 250 kv X-ray set. The spherical " Perspex " pressure vessel has been replaced by hemispherical vessels with flat metal base plates (Fig. 1 and 2). This has enabled dental radiographic plates, 3 $\times$ 4 cm., to be placed outside the chamber in a " Perspex " covered recess in the base plate so as to lie in the vertical axis of the chamber immediately below the tumour. The internal environment of the pressure vessels was controlled as described in the previous paper except that now the thick " Dural " base-plate was heated by electric elements which came under the same thermostatic control as the incoming gases to maintain a temperature of 29° C.

A 2 ml. syringe was attached to the treatment couch in such a way that additional barbiturate could be injected intraperitoneally, when necessary, from outside the chamber. The rat was placed on this couch, which could be moved up and down, in such a way that the tumour lay in the vertical axis of the chamber immediately beneath a hole, 1 inch in diameter, in a fixed lead ring. This lead protected the tissues adjacent to the tumour from radiation, whilst the remainder of the animal was protected by a larger lead ring, with a similar hole, attached to the head of the X-ray set. The two rings collimated the beam, which was continuously monitored.

Tumours were made anoxic by occluding the circulation with a U-shaped " Perspex " clamp which, by means of spring loaded inflatable rubber jaws, maintained an even pressure on the two layers of skin at all points between the tumour and the remainder of the animal. Anoxia of the tumour has been confirmed by N. T. S. Evans using a polarographic oxygen electrode.

---

EXPLANATION OF PLATE

FIG. 1.—Pressure vessel with " Perspex " dome.

FIG. 2.—Pressure vessel : view from above showing tumour beneath the lead collimator.

1

2

Thomlinson and Craddock.

### PROCEDURE

For each experiment a number of different conditions of oxygenation and doses of radiation were chosen, amounting usually to some four to seven modes of treatment, six tumours being allotted to each. The order in which the individual treatments were to be given was taken from a table of random numbers and the tumours were treated in this order as they reached the standard size of 8–10 mm. diameter. This fibrosarcoma, $RIB_5$, remained within the range for one day only.

The animals were anaesthetised with sodium amytal. When the tumour was to be made anoxic for the irradiation, the clamp was applied fifteen minutes beforehand. The tumours were aligned in the axis of the X-ray beam without traction or support except when the clamp was used in the anoxic cases. When animals were to breathe gases other than air, they were placed in the chamber which was flushed, after closure, with twelve changes of the appropriate gas. The pressure was then raised when desired and a period of thirty minutes allowed for equilibration.

The combination of the breathing of oxygen at 4 atm. pressure and anaesthesia, when the latter has depressed the respiration rate below 40 per minute. has occasionally led to a syndrome of respiratory distress. This is characterised by pulmonary oedema and may lead to death or slow recovery from the anaesthesia which is followed by the development of spastic paraplegia.

Radiographs of the tumour were made immediately before and after the irradiation. The ambient temperature, gas pressure, gas flow and the respiration rate of the animal were recorded. After the irradiation the pressure within the chamber was allowed to return to normal over a period of about two minutes.

In the previous report (Thomlinson, 1960) various explanations were considered for the wide deviation of a few irradiated tumours from the mean growth pattern. The use of radiographs in the more recent series has shown this to be due to an occasional failure to irradiate the whole tumour. Where radiographs have indicated such incomplete irradiation the tumours have been excluded from the pooled results. Subsequent examination of the growth of each tumour has shown that this exclusion has eliminated the few growth curves which differed widely from the rest.

Measurements of tumour diameter were made daily or, more recently, on six days out of seven. The mean of the daily measurements for animals receiving the same treatment, together with the standard errors of the mean, have been calculated and the means plotted against time (Fig. 3).

The effect of radiation under different conditions of oxygenation has been compared by using the extra time taken to grow from 9 mm. diameter, the mean size at the time of irradiation, to an arbitrarily chosen diameter of 25 mm. The time at which groups of tumours are taken to have reached this diameter was calculated from the average of the time that each tumour reached the diameters of 21, 23, 25, 27 and 29 mm.

### RESULTS

The mean time taken by 61 unirradiated tumours to grow from 9 to 25 mm. diameter was $6{\cdot}1 \pm 0{\cdot}2$ days. This time has changed little over the period of the experiments as is shown in Table I. This indicates a mean tumour volume

FIG. 3.—Growth curves of tumour $RIB_5$ after single doses of radiation given at average diameter of 9 mm. Tumours measured daily in three dimensions. The curves plotted for the mean of all animals in each group. The numbers of animals are shown in Table V. Standard errors have been omitted for clarity (but see Thomlinson, 1961).

doubling time of 1·35 days. At the size used for irradiation the volume doubles from 8 to 10 mm. in approximately 1 day. At the smallest measurable sizes, between 5 and 7 mm. the volume doubling time is roughly $\frac{3}{4}$ day. The cells have

TABLE I.—*The Rate of Growth of Unirradiated Tumours During the Years* 1960–1964

| Year | No. of animals | Time to grow from 9 mm. to 25 mm. diam. (days) | 95% confidence limits |
|---|---|---|---|
| 1960 | 21 | 5·7 | ±0·2 |
| 1961 | 4 | 5·4 | ±0·6 |
| 1962 | 11 | 6·0 | ±0·2 |
| 1963 | 12 | 6·2 | ±0·2 |
| 1964 | 13 | 7·1 | ±0·2 |

been found to have a cycle time of 14 hours as measured by pulse labelling with tritiated thymidine (Denekamp, 1966, personal communication).

Ancillary procedures, such as the administration of amylobarbitone, the induction of anoxia in tumours by applying the clamp and the breathing of oxygen at high pressure altered the time taken by tumours in unirradiated animals to grow from 9 mm. to 25 mm. by very little (Table II).

TABLE II.—*The Rate of Growth of Unirradiated Tumours Given Ancillary Treatments*

| Procedure | No. of animals | Time to grow from 9 mm. to 25 mm. diam. (days) | 95% confidence limits |
|---|---|---|---|
| Amylobarbitone only | 7 | 6·2 | ±0·2 |
| Amylobarbitone and | | | |
| anoxia for 20 min. | 5 | 6·1 | ±0·4 |
| anoxia for 45 min. | 7 | 5·0 | ±0·2 |
| anoxia for 60 min. | 6 | 5·0 | ±0·2 |
| anoxia for 96 min. | 7 | 5·6 | ±0·2 |
| Amylobarbitone and oxygen breathing at 4 atm. abs. for 20 min. | 5 | 5·7 | ±0·4 |

The response of the tumour to irradiation with air breathing altered in 1960, coinciding with the change of pressure chambers, but changed little during the subsequent period (Table III).

TABLE III.—*The Rate of Growth of Tumours During the Period of the Experiments After a Dose of* 2000 *Rads Whilst Rats were Breathing Air*

| Year | No. of animals | Time to grow from 9 mm. to 25 mm. diam. (days) | 95% confidence limits |
|---|---|---|---|
| 1960 | 7 | 17·8 | ±1·4 |
| 1961 | 5 | 21·0 | ±2·0 |
| 1962 | 12 | 22·0 | ±1·2 |
| 1964 | 5 | 23·0 | ±1·8 |

The results of all single doses of radiation given under different conditions are set out in Table IV. " Oxygenation " refers to the partial pressure of oxygen breathed in atmospheres absolute.

TABLE IV.—*The Rate of Growth of Tumours after Irradiation in Different Conditions of Oxygenation*

| State of oxygenation | Dose K. rads | No. of animals | Time to grow from 9–25 mm. diameter: Days | 95% conf. limits |
|---|---|---|---|---|
| Control | — | 28 | 6·0 | ±0·6 |
| Anoxia | 1 | 6 | 6·4 | ±0·3 |
| | 2 | 6 | 8·6 | ±0·5 |
| | 3 | 11 | 11·8 | ±0·7 |
| | 4 | 10 | 15·8 | ±0·8 |
| | 5 | 12 | 24·7 | ±1·0 |
| | 6 | 5 | 40·0 | ±3·1 |
| Breathing $O_2$ | 1 | 6 | 13·2 | ±0·8 |
| 0·1 atm. | 2 | 7 | 16·4 | ±1·0 |
| (10% $O_2$ in $N_2$) | 4 | 5 | 24·6 | ±1·3 |
| 0·2 atm. (air) | 0·5 | 6 | 8·6 | ±0·5 |
| | 1 | 6 | 10·5 | ±0·5 |
| | 2 | 31 | 21·3 | ±0·8 |
| | 3 | 6 | 24·0 | ±1·9 |
| | 4 | 17 | 26·8 | ±0·7 |
| | 5 | 5 | 36·7 | ±2·4 |
| | 6 | 3 | 42·6 | ±1·6 |
| 1 atm. | 1 | 6 | 11·1 | ±0·3 |
| | 2 | 4 | 20·8 | ±2·0 |
| | 3 | 4 | 30·1 | ±2·0 |
| | 4 | 5 | 33·6 | ±3·5 |
| 2 atm. | 1·5 | 6 | 24·1 | ±0·6 |
| | 2 | 7 | 26·6 | ±2·3 |
| | 3 | 6 | 40·4 | ±1·5 |
| 3 atm. | 1·5 | 4 | 26·3 | ±1·4 |
| | 2·0 | 6 | 30·0 | ±3·5 |
| | 2·5 | 3 | 30·3 | ±3·2 |
| | 3·0 | 4 | 39·3 | ±3·4 |
| 4 atm. | 0·4 | 6 | 8·2 | ±0·5 |
| | 1·0 | 6 | 14·1 | ±0·6 |
| | 1·5 | 6 | 23·5 | ±1·2 |
| | 2·0 | 6 | 28·2 | ±0·9 |
| | 3·0 | 6 | 30·8 | ±2·8 |
| | 4·0 | 7 | 35·7 | ±3·1 |

After irradiation there was a second wave of delay in the growth of the tumours (Fig. 3). This is also shown by the few tumours which have been irradiated when 13 mm. in diameter (Fig. 4). The time after irradiation at which the minimal rate of growth occurs during this second wave of delay is shown in Table V. Table V also includes the interval between this time and the time at which the tumours, after the initial period of regression had grown again to the size of 9 mm. or 13 mm. diameter at which they had been irradiated.

The growth rate of tumours, after they had reached a diameter of 20 mm. is not the same after irradiation as that of untreated tumours. The times taken for growth from the diameter of 21 mm. to 29 mm. have been used to compare these growth rates after the different treatments and are shown in Table VI.

FIG. 4.—Growth curves of tumour $RIB_5$ after irradiation at 13 mm. diameter.

### DISCUSSION

*Part* 1. *Modifications of the gross response to irradiation resulting from changes of concentration of respired oxygen and their relation to the cell-killing effect of radiation.*

A comparison of the effect of different treatments has been made by plotting dose-effect curves from the times, shown in Table IV, taken by tumours to grow from the size used for irradiation to 25 mm. diameter (Fig. 5).

Tumour $RIB_5$ has been examined histologically and the relative volumes of intact and necrotic tissue throughout the tumours computed from serial sections. In different specimens of tumours of the same size these proportions are constant. The tumours may, therefore, tentatively be assumed to contain the same number of cells capable of division. The parameter " time to grow " may consequently be taken as a measure of the rate of increase of the cell population. Because of this, if it could be assumed that the fraction of tumour cells proliferating and their cell-cycle time were the same before and after irradiation (see Part 2 of this discussion), the ordinate " time to grow " used in Fig. 5 would be a measure of the number of cells from which the irradiated tumours grew again and so would be analogous to that of " surviving fraction " on a logarithmic scale as used in cell survival experiments.

TABLE V.—*The Time of the Second Wave of Delay in Growth of Tumours after Irradiation*

| State of oxygenation | Dose K. rads | Day after irradiation of minimal growth rate, in second wave of delay | Time between re-growth to size at irradiation and second wave of delay |
|---|---|---|---|
| Anoxia | 3·0 | 9·5 | 7·0 |
| | 4·0 | 12·5 | 6·5 |
| | 5·0 | 17·5 | 5·5 |
| | 6·0 | 23·5 | 4·5 |
| Breathing $O_2$ | 2·0 | 10·5 | 8·0 |
| 0·1 atm. | 4·0 | 14·5 | 8·5 |
| (10% $O_2$ in $N_2$) | | | |
| 0·2 atm. (air) | 1·0 | 8·5 | *6·5* |
| | 2·0 | 11·5 | *8·0* |
| | 3·0 | 15·5 | 8·0 |
| | 4·0 | 18·5 | 8·0 |
| | 5·0 | 27·5 | 7·5 |
| | 6·0 | 28·5 | 5·0 |
| 1 atm | 2·0 | 10·0 | 5·0 |
| | 3·0 | 19·0 | 10·0 |
| | 4·0 | 21·0 | 9·0 |
| 2 atm. | 1·5 | 14·5 | 6·5 |
| | 2·0 | 15·5 | 6·5 |
| | 3·0 | 29·0 | 9·0 |
| 3 atm. | 1·5 | 15·5 | 6·5 |
| | 3·0 | 26·5 | 7·5 |
| 4 atm. | 1·0 | 9·5 | 5·5 |
| | 1·5 | 14·5 | 6·5 |
| | 2·0 | 19·5 | 9·0 |
| | 3·0 | 22·0 | 8·0 |
| | 4·0 | 28·5 | 8·5 |
| Tumours irradiated at 13 mm. | 2 | 11·5 | 6·5 |
| diam. 0·1 atm. | 4 | 19·5 | 8·0 |
| (air) | | | |

From these curves it can be seen that in each of the conditions, other than anoxia, in which the tumours were irradiated the points obtained fall approximately on a combination of two theoretical curves : the first, a theoretical curve for full oxygenation, obtained by dividing the doses given anoxically by a constant factor of 3·4 found by trial and error ; the second, anoxic curves drawn from higher points of origin on the ordinate chosen according to the experimental results. This treatment has demonstrated some similarities with the lethal effect of radiation on cells.

One characteristic of the effect of radiation in killing cells is that changes in the concentration of oxygen around them at the time of irradiation often modifies the dose required to produce a given effect, that is to say, any given effect obtained with one concentration of oxygen can be obtained with another by changing the dose by a factor solely dependent on the two concentrations (Read, 1952 ; Alper, 1966). The maximum factor relating dose-effect curves for anoxia and full oxygenation has generally been found to lie between 2·3 and 3·0 for mammalian cells.

Irradiation of a population containing both well-oxygenated and anoxic cells results in a composite survival curve (Gray, 1961 ; Powers and Tolmach, 1963). The initial part of this corresponds to that for a wholly oxygenated population and the final part for a wholly anoxic population. The anoxic component can be extrapolated to a point on the ordinate corresponding to the proportion of

FIG. 5.—Dose-effect curves for tumour $RIB_5$. The effect is represented by the time taken to grow from the diameter when irradiated, i.e. 9 mm. to 25 mm. diameter. Unirradiated tumours take six days. The delay induced by radiation is the result of the reduction of numbers of clonogenic cells, altered growth of survivors, and damage to the vascular stroma.

TABLE VI.—*The Rate of Growth of Tumours Between* 21 *and* 29 *mm. Diameter after Irradiation*

| State of oxygenation | Dose K. rads | No. of animals | Time to grow from 21–29 mm. diam. Days | 95% conf limit |
|---|---|---|---|---|
| Control | — | 28 | 2·6 | ±0·6 |
| Anoxia | 1 | 6 | 2·7 | ±0·4 |
| | 2 | 6 | 2·8 | ±0·2 |
| | 3 | 11 | 4·2 | ±0·3 |
| | 4 | 10 | 4·4 | ±0·5 |
| | 5 | 12 | 4·6 | ±0·3 |
| | 6 | 5 | 4·5 | ±0·4 |
| Breathing $O_2$ | 1 | 6 | 4·1 | ±0·1 |
| 0·1 atm. | 2 | 7 | 5·7 | ±0·4 |
| (10% $O_2$ in $N_2$) | 4 | 5 | 5·8 | ±1·1 |
| 0·2 atm. (air) | 0·5 | 6 | 3·7 | ±0·3 |
| | 1 | 6 | 4·2 | ±0·7 |
| | 2 | 31 | 5·7 | ±0·3 |
| | 3 | 6 | 4·7 | ±0·7 |
| | 4 | 17 | 4·6 | ±0·5 |
| | 5 | 5 | 4·2 | ±0·7 |
| | 6 | 3 | 4·0 | ±0·6 |
| 1 atm | 1 | 6 | 4·6 | ±0·3 |
| | 2 | 4 | 6·8 | ±1·3 |
| | 3 | 4 | 3·6 | ±0·4 |
| | 4 | 5 | 3·6 | ±0·7 |
| 2 atm. | 1·5 | 6 | 4·8 | ±0·1 |
| | 2 | 7 | 5·9 | ±1·2 |
| | 3 | 6 | 4·4 | ±0·5 |
| 3 atm. | 1·5 | 4 | 5·0 | ±0·5 |
| | 2·0 | 6 | 4·4 | ±0·8 |
| | 2·5 | 3 | 5·8 | ±0·3 |
| | 3·0 | 4 | 6·3 | ±0·4 |
| 4 atm. | 0·4 | 6 | 2·4 | ±0·2 |
| | 1·0 | 6 | 5·0 | ±0·4 |
| | 1·5 | 6 | 6·0 | ±0·6 |
| | 2·0 | 6 | 5·0 | ±1·0 |
| | 3·0 | 6 | 5·2 | ±0·6 |
| | 4·0 | 7 | 4·0 | ±0·4 |

anoxic cells in the whole population (Fig. 6). Plotted in this way the anoxic components are parallel only when the cell survival curve is exponential. Where survival curves were continuously bending (Barendsen, 1961 ; Sinclair and Morton, 1965) or where dose-effect curves derived from counting total cell populations were continuously bending (Fowler, 1966) the anoxic components would be of the same shape but equidistant only in the direction of the ordinate (Fig. 7).

As a result of changes in the proportion of anoxic cells extrapolation of the anoxic components of survival curves would meet the ordinate at different points. Such changes can be induced in the cell population of tumours by altering the oxygen content of the inspired gas. This has been demonstrated by the cell survival curves obtained after the irradiation of lymphomas in mice (Powers and Tolmach, 1964).

The gross response of tumour $RIB_5$ to irradiation in other than anoxic conditions results in dose-effect curves which are composite (Fig. 5). The initial portion is a curve derived from the " anoxic curve " by modifying the dose. It therefore appears that the gross response of this tumour to radiation is modified by oxygen in exactly the same manner as is the survival of cells after irradiation.

It is tempting to conclude from this that the gross response is a manifestation of a cellular response and that, since the neoplastic cells of the tumour far outnumber all others, it is a response of these cells alone. However there are reasons, discussed in Part 2, for believing that other factors modify the growth of surviving neoplastic cells making the proportion of them which survive but one of the factors involved in the gross response, albeit the dominant one.

If the survival of the tumour cells were the only factor involved it would be possible to construct cell survival curves from the gross response. Curves have

FIG. 6.—Theoretical cell survival curves for anoxic and oxygenated cells and a mixed population containing 1% anoxic cells.

been drawn as if this were so by using the growth curve of the unirradiated tumour, the diameter being plotted on a logarithmic scale (Fig. 8). Growth is exponential when the tumour is small, but with the development of massive central necrosis, in larger tumours, the rate diminishes. From points in time at which irradiated tumours reach a particular diameter this curve can be used to extrapolate to a diameter on the day of radiation from which a hypothetical unirradiated tumour would have grown to the same point. The volume of such hypothetical tumours bears the same relation to that of the actual tumours when they were irradiated as does the number of clonogenic cells which survive to that which was irradiated. By calculation of these volumes a series of cell survival curves can therefore be constructed (Fig. 9). These cannot be accurate because of the assumptions on which they are based, but they are not dissimilar to other published curves, for example, those for the Ehrlich mouse ascites tumour cells (Hornsey and Silini, 1961).

These findings warrant the conclusion that, whilst the animal is breathing air, the tumour does contain cells which are protected from radiation injury by anoxia

FIG. 7.—Theoretic survival curves obtainable by counting total number of cells in all irradiated clones at a chosen time as a proportion of progeny of unirradiated clones after, say, ten cell divisions (Whitmore and Till, 1964 ; Fowler, 1966).

FIG. 8.—Growth curve of untreated tumour $RIB_5$ with diameter plotted on logarithmic ordinate to show period of exponential growth.

and which are able to proliferate in the environment which develops in the tumour after single doses of radiation. The proportion of such cells in the population can be changed by altering the concentration of oxygen in the respired gas. The position of the dose-effect curve obtained with any particular concentration, relative to those obtained under totally anoxic conditions and with full oxygenation, is a measure of this proportion.

FIG. 9.—Approximate cell-survival curves of tumour $RIB_5$. The times were determined for tumours to grow to diameters of 20 mm. and 30 mm. after irradiation. From these, extrapolation using the curve in Fig. 8 gave the diameter on the radiation day of hypothetical tumours which, without radiation, would have grown to 20 mm. and 30 mm. in the observed times. The volume of such theoretical tumours as a fraction of the volume of the actual tumours irradiated would represent the surviving fraction of cells, were the killing of clonogenic cells the only effect of the irradiation. The boundaries of the shaded areas represent the curves obtained by using the two diameters of 20 mm. and 30 mm. respectively. The times from which these curves have been deduced are also affected by the radiation injury to the vascular stroma and the altered growth rate of irradiated tumours. No allowance has been made for this.

*Part 2. Changes in tumour growth-rate after irradiation*

After single doses of radiation tumour $RIB_5$ enlarges for about two days, regresses for a period dependent on the size of the dose, then grows again. The early changes are under investigation. The curves of the later stage of growth appear to depart from a curve for complete regression of the tumour (Fig. 10 and 3) at different points in time. Once growth has started again its progress is not likely to be influenced by the factors effecting regression.

Two aspects of the subsequent growth merit discussion : first, a second wave of delay or regression seen in the growth curves ; and second, the different growth rates of tumours at the larger sizes.

The second wave of delay of growth (Fig. 3 and 4) occurred after irradiation in all conditions of oxygenation and is apparent after most doses, though it is

FIG. 10.—Curves of the growth of untreated tumours and the regression of 21 tumours in animals which had been cured.

less evident when tumours had been irradiated under anoxic conditions. It occurred later in time and is of greater length as the dose is increased. However, the difference in time between that at which the groups reached the size at which they had been irradiated and that during the second wave when the growth rate was least appeared nearly constant ; $7 \cdot 0 \pm 0 \cdot 3$ days for 25 groups of tumours radiated when 9 mm. diameter and $7 \cdot 25 \pm 0 \cdot 8$ days for 2 groups of tumours radiated when 13 mm. diameter. At first sight, therefore, it appeared that this time interval was independent of the size of the tumour and the condition of oxygenation when it was irradiated and of the dose given, but the exact relationship of this time to the dose given will be considered with the different growth rates.

Although there is evidence of circulatory failure and stagnation of blood in the capillaries of this tumour, thrombosis of capillaries in tumours which have not been irradiated and in the period immediately following the irradiation is almost unknown. In the period of the second wave of delayed growth up to half the capillaries in the tumours examined have been found to be thrombosed.

These observations can be explained by a hypothesis. Endothelial cells of capillaries were irradiated but so long as there was no call for proliferation no injury was manifest. When the tumours, after regressing for a while, grew up to and beyond the size at which they had been irradiated cell-division in the endothelial cells was stimulated and this led to cell-death, capillary thrombosis and a new wave of necrosis of tumour cells. This hypothesis is the subject of further investigation.

When tumours grew again after irradiation, the rate of growth was not the same as that of untreated tumours. This was most clearly seen in the larger tumours after the second wave of delay (Fig. 3). The time they took to grow from 21 mm. to 29 mm. diameter has been taken as a measure of growth rate (Table VI). Untreated tumours took approximately 2·6 days. After irradiation under anoxic conditions the time taken to grow between these two sizes lengthened as the dose was increased. This was also true when the animals had breathed 10% oxygen in nitrogen during the irradiation, but with the breathing of air or pure oxygen the time lengthened and then shortened again. This confused situation was somewhat clarified if the time taken by tumours to grow to 25 mm. (Table IV)—itself a measure of the effectiveness of the dose—was used rather than the dose itself. This time has been related to the time taken to grow from 21 mm. to 29 mm. diameter (Fig. 11). Whatever the condition of oxygenation, all points lie, with considerable scatter, about a single curve. With the increasing effectiveness of radiation the time taken to grow from 21 mm. to 29 mm. rises and falls again, that is to say, the growth rate was at first decreased and then increased again.

An explanation of this phenomenon may lie in the alteration of the growth-rate of the progeny of surviving cells in a manner analogous to that causing the development of microcolonies from irradiated cells *in vitro* (Sinclair, 1964 ; Nias *et al.*, 1965). In such experiments the growth rate of clones derived from single unirradiated cells varies over a small range. This range is extended as the dose of radiation rises because an increasing proportion of the clones grows more slowly. The slower growth rate appears to be due either to a lengthening of the cell cycle time or to an increase of the proportion of the cells dying within the clones, or a combination of the two. The growth of a tumour reflects the mean rate of population increase at any given time, so that, if the clonogenic survivors of irradiation were affected in the same way as cells in culture, the growth rate of the tumour would be reduced. However, this mean represents a mixed population, part of which is growing more rapidly. The growth rate of the tumour would consequently change with time toward the rate of the faster growing cells. Measurements of the growth-rate of tumour $RIB_5$ (Fig. 11) have been made on populations of approximately the same size but at different times after irradiation. The more effective the radiation has been, the smaller the number of survivors which must, therefore, go through more generations to produce a population of this size. The return toward the faster growth-rate probably results from the proportion of survivors with a faster growth rate having a longer time in which to outnumber those with a slower rate.

In the experiments in which microcolonies have been observed, the effect of irradiation can be expressed as the total number of cells in all clones rather than as the number of clones each derived from one surviving cell. Where the proportion of small clones increases with dose, curves relating the total number of cells in the population at some chosen time after irradiation to the dose given would bend downward with increasing dose even if the corresponding number of survivors

FIG. 11.—Points relating the time taken by tumours to grow from 21 mm. to 29 mm. diameter as a measure of growth rate related to the time taken from 9 mm. to 25 mm. as a measure of the affect of radiation (the relation between the latter time and dose is given in Table IV and Fig. 5).

were a straight exponential line (Fowler, 1966). If the time taken by the irradiated tumours to reach a diameter of 25 mm. were a measure of the time it takes the progeny of surviving cells to reach a certain number, curves relating this time to dose would bend upward with increasing dose. That they do so (Fig. 5) lends support to the notion of a close relationship between the gross response of the tumour and the survival of its clonogenic cells.

Some correlation can be found between the slower growth rate of the tumours seen between the sizes of 21 mm. and 29 mm. diameter and the time taken for the second wave of delay in growth to develop after the tumours have regrown to the size at which they were irradiated. This suggests that the mechanism of the delay and the capillary thrombosis which is its immediate cause do indeed result from enlargement of the tumour.

The exact part played in the growth of tumours after irradiation by the large number of dead and dying cells amongst the survivors (Révész, 1958) has not been assessed, neither has the influence of any possible immune reaction. It is difficult to see how either could have produced the effects observed.

### SUMMARY AND CONCLUSION

The gross response of tumour $RIB_5$ appears to be made up of the compound effect of radiation in killing clonogenic cells of the tumours, in altering the growth rate of survivors and in causing damage to the vascular stroma. The response to changes in concentration of respired oxygen suggest that the effect on cell killing dominates the picture. This is perhaps to be expected in this tumour which is anaplastic, and which is so rapidly enlarging as to indicate that most of its cells are proliferating. However, even the oxygen enhancement ratio for the gross response of the tumour appears to be considerably greater than that of its clonogenic cells tested *in vitro* (McNally, 1966, personal communication). The difference may be due to the effects on the capillaries. Caution is therefore necessary in deducing the effects of radiation in killing cells from the gross response of tumours or in inducing hypotheses about the effects of radiation on whole tumours from cell survival observations alone.

We should like to thank Miss Tikvah Alper and Dr. G. J. Popják for their support and advice, Professor J. F. Fowler, Sir Oliver Scott and Dr. H. B. Hewitt for valuable discussion and advice, Mr. L. Tout and the staff of the animal house for the production of large numbers of animals, Mr. A. Roth and the staff of the mechanical workshop for the production of the pressure vessels, and the Plastics Division of the Imperial Chemical Industries, Ltd., who kindly developed and gave the " Perspex " domes. We are also indebted to almost all the other members of this unit for their help.

### REFERENCES

ALPER, T.—(1966) In : ' Modern Trends in Radiotherapy '. Edited by T. J. Deeley, London (Butterworths), p. 1–33. In press.

BARENDSEN, G. W.—(1961) In : ' The Initial Effects of Ionising Radiations on Cells '. London and New York (Academic Press), pp. 183–194.

FOWLER, J. F.—(1966) In : ' Current Topics in Radiation Research '. Edited by M. Ebert and A. Howard. Amsterdam (North Holland Publishing Co.), Vol. 2, pp. 312–364.

GRAY, L. H.—(1961) *Am. J. Roentg.*, **85**, 803.

HORNSEY, S. AND SILINI, G.—(1961) *Int. J. Radiat. Biol.*, **4**, 135.

NIAS, A. H. W., GILBERT, C. W., LAJTHA, L. G. AND LANGE, C. S.—(1965) *Int. J. Radiat. Biol.*, **9**, 275.

POWERS, W. E. AND TOLMACH, L. J.—(1963) *Nature, Lond.*, **197**, 710.—(1964) *Radiology*, **83**, 328.

READ, J.—(1952) *Br. J. Radiol.*, **25**, 154.

RÉVÉSZ, L.—(1958) *J. natn. Cancer Inst.*, **20**, 1157.

SINCLAIR, W. K.—(1964) *Radiat. Res.*, **21**, 584.

SINCLAIR, W. K. AND MORTON, R. A.—(1965) In : ' Cellular Radiation Biology '. Baltimore, U.S.A. (Williams and Wilkins), pp. 418–422.

THOMLINSON, R. H.—(1960) *Br. J. Cancer*, **14**, 555.—(1961) In : ' Fundamental Aspects of Radiosensitivity '. *Brookhaven Symp. Biol.*, No. 14.

WHITMORE, G. F. AND TILL, J. E.—(1964) *A. Rev. nucl. Sci.*, **14**, 347.